# CCL18 and EGF May Serve as Potential Prognostic Biomarkers and Therapeutic Targets for Human Breast Cancer

**DOI:** 10.1155/ijbc/8856457

**Published:** 2025-07-14

**Authors:** Sm Faysal Bellah, Fatema Akter Sonia, Md. Razowanul Ferdous, Olanrewaju Ayodeji Durojaye, Md. Robiul Islam

**Affiliations:** ^1^Department of Pharmacy, Manarat International University, Dhaka, Bangladesh; ^2^Department of Pharmacy, Bangabandhu Sheikh Mujibur Rahman Science & Technology University, Gopalganj, Bangladesh; ^3^Research and Development Division, The ACME Laboratories Ltd., Dhaka, Bangladesh; ^4^Drug Discovery and Biotechnology Unit, Lion Science Park, University of Nigeria, Nsukka, Nigeria; ^5^Department of Chemical Sciences, Coal City University, Emene, Enugu State, Nigeria

**Keywords:** breast cancer, CCL18, EGF, overall survival

## Abstract

**Background:** Breast cancer (BRCA) remains a leading cause of cancer-related mortality among women. CCL18 and EGF are implicated in tumor biology; however, their roles in BRCA remain partly defined. This study investigates their expression profiles, immune associations, prognostic relevance, epigenetic regulation, and molecular networks.

**Methods:** Expression data from TCGA, UALCAN, and GSCA were analyzed to compare CCL18 and EGF levels in BRCA and normal tissues. Immune infiltration was assessed using TIMER, while survival analyses were performed via Kaplan–Meier plotter and TCGA subcohorts, including menopausal status. Promoter DNA methylation was examined using UALCAN. Gene correlation networks and protein–protein interactions were assessed using UALCAN and STRING.

**Result:** CCL18 was significantly upregulated in BRCA tissues, while EGF showed no consistent increase compared to normal tissue. Both genes were strongly correlated with immune cell infiltration. High CCL18 and EGF expression was associated with reduced relapse-free survival in BRCA. Promoter regions of both genes exhibited reduced DNA methylation, supporting their elevated expression in tumors. Interaction analyses revealed distinct immune- and signaling-related gene and protein networks.

**Conclusion:** CCL18 shows strong prognostic and immunological relevance in BRCA, while EGF appears to play a broader oncogenic role. Hypomethylation of both genes may drive their aberrant expression and involvement in tumor progression.

## 1. Introduction

Breast cancer (BRCA) is one of the most common forms of cancer and the fifth leading cause of cancer mortality worldwide. As the primary cause of cancer incidence in 2020, BRCA surpassed lung cancer with a projected 2,261,419 cases (11.7% of all sites) new cases and 684,966 (6.9% of all sites) deaths globally. It ranks first for incidence in the great majority of nations (159 out of 185) and for mortality in 110 countries, accounting for 1 in 4 cancer cases and 1 in 6 cancer deaths among females [1]. The highest incidence rates (> 80 per 100,000) are found in Australia/New Zealand, Western Europe (Belgium has the highest incidence in the world), Northern America, and Northern Europe, while the lowest incidence rates (< 40 per 100,000) are found in Central America, Eastern and Middle Africa, and South Central Asia. Developed countries have incidence rates that are 88% higher than those of developing countries (55.9 and 29.7 per 100,000, respectively). However, women living in developing countries have 17% higher mortality rates compared with women in developed countries (15.0 and 12.8 per 100,000, respectively) because of high fatality rates, with the highest mortality rates found in Melanesia, Western Africa, Micronesia/Polynesia, and the Caribbean (Barbados has the world's highest mortality) [1, 2]. BRCA was the commonest cancer among the female patients in Bangladesh according to morbidity and mortality [3].

The CC chemokine family includes the small cytokine known as chemokine (C-C motif) ligand 18 (CCL18) which is predominantly produced by monocyte-derived cells with M2 phenotype [4]. Its production can be upregulated by interleukins (IL-4, IL-10, and IL-13), which are cytokines that favor a Type 2 immune response. Besides other consequences, higher concentrations of CCL18 in the serum or the tumor are linked to a worse prognosis for cancer patients [5]. High levels of CCL18 produced by M2 macrophages have been detected in chronic inflammatory and fibrotic conditions, including Gaucher's disease and rheumatoid arthritis [6]. Furthermore, CCL18 is consistently expressed by macrophages infiltrating various tumors, including ovarian, gastric, BRCAs, and glioma [7]. In particular, BRCA is the condition in which CCL18 is most evidently involved. CCL18 derived from tumor-associated macrophages (TAMs) plays a critical role in promoting BRCA metastasis via its receptor, PITPNM3 [8]. CCL18 may function as an immunosuppressive cytokine in BRCAs by generating immunosuppressive dendritic cells, macrophages, and T regulatory cells, as well as driving effector T cells to abolish their anticancer capabilities and immune system [9]. Breast TAMs secrete CCL18, which downregulates miR-98 and miR-27b, enhancing angiogenesis, adhesion, and migration of cancer cells. CCL18 also promotes tumor growth and metastasis by inducing epithelial–mesenchymal transition through the PI3K/Akt/GSK3/Snail signaling pathway [10]. It is known that when carcinoma-associated fibroblasts and BRCA cells are cocultured, CCL18 production increases, promoting the invasion and metastasis of BRCA cells [11].

Epidermal growth factor (EGF), a 53-amino acid peptide, regulates cell proliferation and differentiation. Its receptor, EGFR, is commonly overexpressed in ER-negative BRCA cells, primary tumors, and metastases, highlighting its role in cancer progression [12]. Both normal and malignant mammary epithelial cells produce EGF-related peptides such as TGF-*α*, amphiregulin (AR), heregulin (HRG), and cripto-1 (CR-1). However, cancerous cells typically exhibit higher expression levels of TGF-*α*, AR, and CR-1, suggesting their significant involvement in BRCA development [13]. EGFR activation enhances BRCA cell migration, proliferation, and resistance to apoptosis. Approximately 25% of metaplastic BRCA cases are associated with EGFR gene involvement. While activating EGFR mutations are common in lung and CNS tumors, they are rare in BRCA, where overexpression typically occurs through alternative mechanisms [14]. In vitro and in vivo studies show that macrophages enhance cancer cell invasion and migration by releasing cytokines and chemokines regulated by EGF and CCL18 or by remodeling the extracellular matrix through protease activity, increasing chemoattractant availability [15, 16].

HER2 and BRCA1 are widely recognized BRCA biomarkers with critical roles in oncogenesis and therapy. HER2 is a receptor tyrosine kinase which is overexpressed in 15%–20% of BRCAs and drives tumor aggressiveness but also allows effective targeting with monoclonal antibodies like trastuzumab [17]. BRCA1 is a DNA repair gene, when it mutated, predisposes the individuals to early-onset BRCA, particularly triple-negative subtypes [18, 19]. However, these biomarkers do not fully account for tumor microenvironment interactions. CCL18, secreted by M2-type macrophages, promotes immune evasion and metastasis through epithelial–mesenchymal transition. EGF, binding to EGFR, facilitates proliferation and resistance to apoptosis, especially in ER-negative tumors. These molecules reflect dynamic stromal–immune signaling rather than static genetic mutations, offering insights into tumor behavior beyond current markers. Studying CCL18 and EGF may thus expand our understanding of BRCA progression and identify new therapeutic vulnerabilities.

BRCA remains one of the most prevalent and deadly malignancies affecting women worldwide, necessitating the continuous search for novel biomarkers to improve diagnosis, prognosis, and treatment strategies. Although numerous genes and signaling pathways have been linked to BRCA development, the specific involvement and interplay of chemokine ligand CCL18 and EGF in BRCA biology remain insufficiently characterized. In this study, we employed publicly accessible computational biology tools to investigate the expression profiles, molecular associations, and prognostic implications of CCL18 and EGF in BRCA. The goal was to uncover their potential contributions to BRCA risk and progression, offering new insights into their utility as biomarkers or therapeutic targets.

## 2. Methods

### 2.1. Analysis of Gene Expression and Immune Infiltration Profile Using The Cancer Genome Atlas (TCGA) Cancer Data

TIMER2.0 (http://timer.cistrome.org/) is a comprehensive web tool designed to analyze and visualize immune cell infiltration across various cancer types using data from TCGA. It provides multiple analytical modules to explore the relationships between gene expression and immune infiltration. Specifically, the Gene_DE module allows users to compare the expression levels of a selected gene between tumor tissues and their adjacent normal counterparts across all TCGA cancer types. The TCGA dataset integrated into TIMER2.0 includes a wide range of cancer types along with corresponding normal tissue data, enabling detailed investigations into gene expression patterns and immune microenvironment dynamics in cancer [20, 21].

### 2.2. Assessment of CCL18 and EGF Expression in BRCA

To analyze the expression levels of CCL18 and EGF in breast invasive carcinoma (BRCA), we utilized the Gene Expression Profiling Interactive Analysis (GEPIA2) web tool (http://gepia2.cancer-pku.cn) and Gene Set Cancer Analysis (GSCA) (https://guolab.wchscu.cn/GSCA/#/expression). GEPIA2 is an advanced interactive platform designed to provide comprehensive analyses of gene expression data from TCGA. This tool allows for the examination of gene expression patterns across various cancer types, including BRCA. We accessed TCGA's BRCA dataset to evaluate the expression profiles of CCL18 and EGF and performed correlation analysis to explore potential associations between these two genes [22]. GSCA was also used for the analysis of CCL18 and EGF expression in different stages of breast invasive cancer [23].

### 2.3. Survival Analysis

The Kaplan–Meier plotter (http://www.kmplot.com/), a database containing both clinical and gene expression data, was used to assess the prognostic significance of CCL18 and EGF mRNA expression in BRCA [24]. The BRCA database included the relapse-free survival (RFS) of 4929 patients and the overall survival (OS) of 1879 patients. In summary, samples were split into two cohorts based on the median expression of CCL18 and EGF (high vs. low expression) after the genes were uploaded into the database. This allowed for the creation of Kaplan–Meier survival plots, where the number-at-risk was displayed beneath the main plot. The website provided the estimated log-rank *p* value and hazard ratio (HR) with 95% confidence intervals. A *p* value of less than 0.05 was deemed statistically significant. “Array quality control” was used for this investigation in order to “exclude biased arrays.” Using the survival of TCGA data collected by the UALCAN (http://ualcan.path.uab.edu/) tool, the survival probability of patients with BRCA was also examined based on the expression of CCL18 and EGF with menopause status [21, 25].

### 2.4. Assessment of Promoter Methylation Profile of CCL18 and EGF in Breast Invasive Cancer

To evaluate the DNA methylation status of the CCL18 and EGF promoters in breast invasive carcinoma, we utilized the UALCAN web tool (http://ualcan.path.uab.edu/). UALCAN is a user-friendly platform that allows for the exploration of various omics data, including DNA methylation levels, in relation to cancer. Using data from TCGA, UALCAN enables the assessment of promoter methylation patterns for genes of interest across different cancer types. For this analysis, we focused on the CCL18 and EGF genes in breast tumor samples, comparing their promoter methylation profiles between tumor and normal tissues. This approach provided insights into the potential epigenetic regulation of CCL18 and EGF in breast invasive carcinoma [21, 25].

### 2.5. Gene Correlation Analyses

To identify genes that interact or coexpress with CCL18 and EGF, we performed correlation analysis using RNA expression values. This was done through Pearson correlation analysis, which assesses the linear relationship between the expression levels of two genes. Using UALCAN, we analyzed the expression data of all protein-coding genes from TCGA, calculating Pearson correlation coefficients for each gene pair [25]. A coefficient of 0.3 or higher was considered indicative of a positive correlation, while a coefficient of −0.3 or lower was considered a negative correlation. To ensure robustness, genes with extremely low expression levels (median transcript per million, TPM < 0.5) were excluded from the analysis, as they are unlikely to contribute meaningful correlation data. This approach allowed us to pinpoint genes that are potentially linked to CCL18 and EGF in breast invasive carcinoma.

### 2.6. Protein–Protein Interaction (PPI) Network Construction

The STRING database (http://string-db.org/) is an essential tool for constructing PPI networks, offering a comprehensive integration of both experimentally validated and computationally predicted interactions across various species. For this study, we focused exclusively on interactions relevant to *Homo sapiens* to ensure specificity. To increase the reliability of the results, we applied a high-confidence threshold of 0.9, which filters interactions with lower confidence scores. Using this stringent criterion, we identified a significant PPI between CCL18 and EGF, highlighting its potential relevance in the context of BRCA. This approach allowed us to explore the functional connections between these proteins within the human proteome [26].

### 2.7. Statistical Analysis

The significance levels are represented as follows: ns indicates not significant, ∗ corresponds to a *p* value < 0.05, ∗∗ represents a *p* value < 0.01, and ∗∗∗ denotes a *p* value < 0.001. Log-rank *p* values, along with HRs and their 95% confidence intervals, were calculated, with *p* values below 0.05 considered statistically significant.

## 3. Results

### 3.1. Differential Expression Analysis of CCL18 and EGF Across TCGA Tumors

To investigate the expression profiles of CCL18 and EGF across multiple cancer types, we performed a comprehensive differential expression analysis using data from TCGA. Gene expression levels in tumor tissues were compared to those in matched or adjacent normal tissues using the Wilcoxon rank-sum test, a robust nonparametric method appropriate for assessing expression differences in heterogeneous datasets.

**Figure 1:**
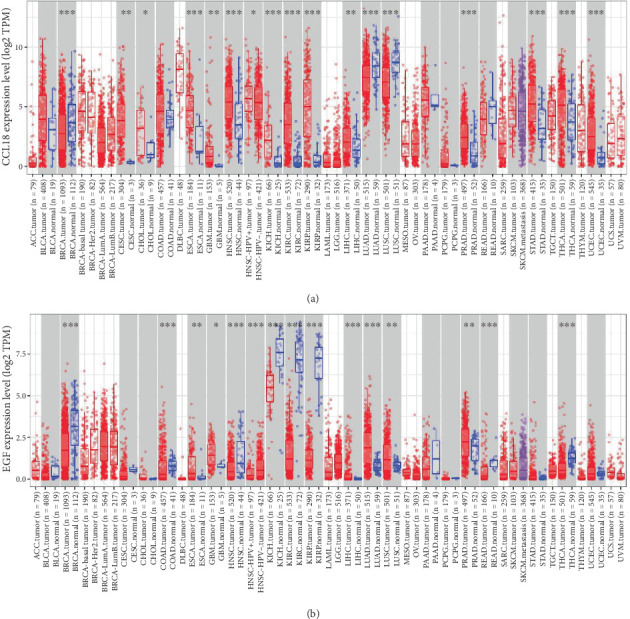
Differential expression of CCL18 and EGF across TCGA cancer types. Boxplots display the gene expression levels of (a) CCL18 and (b) EGF across various TCGA cancer types, comparing tumor tissues to their corresponding adjacent normal tissues. The analysis was performed using the Gene_DE module of the TIMER2.0 platform, which enables differential expression analysis across all TCGA tumor types. Expression distributions are shown for each cancer type where matched normal tissue data are available, highlighted in gray columns. Statistical comparisons were conducted using the Wilcoxon test, with significance levels indicated as follows: *p* < 0.05 (∗), *p* < 0.01 (∗∗), and *p* < 0.001 (∗∗∗). This analysis identifies cancer-specific upregulation or downregulation of CCL18 and EGF across different tumor types.

Our analysis revealed that CCL18 was significantly upregulated in a broad range of tumor types, including breast invasive carcinoma (basal and HER2-enriched subtypes), cervical squamous cell carcinoma and endocervical adenocarcinoma (CESC), cholangiocarcinoma, esophageal carcinoma, glioblastoma multiforme, head and neck squamous cell carcinoma, kidney chromophobe, kidney renal clear cell carcinoma, kidney renal papillary cell carcinoma, liver hepatocellular carcinoma, pancreatic adenocarcinoma, stomach adenocarcinoma, thyroid carcinoma, and uterine corpus endometrial carcinoma (Figure 1a). In contrast, EGF exhibited significant differential expression in a more limited subset of cancers, specifically esophageal carcinoma, glioblastoma multiforme, liver hepatocellular carcinoma, lung adenocarcinoma (LUAD), lung squamous cell carcinoma, and pancreatic adenocarcinoma (Figure 1b).

These findings indicate that both CCL18 and EGF are differentially expressed in several tumor types, suggesting their potential roles as pan-cancer biomarkers. Notably, CCL18 expression was particularly elevated in BRCA subtypes, whereas EGF tended to be downregulated. The expression patterns of both genes in various cancers suggest their involvement in tumorigenesis, although their significance may be context-dependent and influenced by tumor-specific microenvironments.

Despite their broad expression across cancer types, these genes were not significantly altered in all tumors examined, indicating variability in their biological roles. Taken together, our findings highlight the relevance of CCL18 and EGF in cancer biology and underscore their potential as biomarkers, where their expression changes may have diagnostic and therapeutic implications.

### 3.2. Expression Patterns of CCL18 and EGF in BRCA Subtypes and Clinical Stages

Building upon the observed differential expression of CCL18 and EGF across multiple tumor types, we next focused on evaluating their expression specifically within the context of BRCA. This analysis was aimed at determining whether the expression of these genes correlates with disease progression or molecular subtype within the TCGA BRCA cohort.

When stratified by clinical stage, neither CCL18 nor EGF displayed significant variation in expression levels across Stages I–IV (Figures S1A–S1D). This lack of stage-dependent differential expression suggests that while these genes are involved in BRCA biology, their expression is not tightly linked to the clinical progression of the disease.

**Figure 2:**
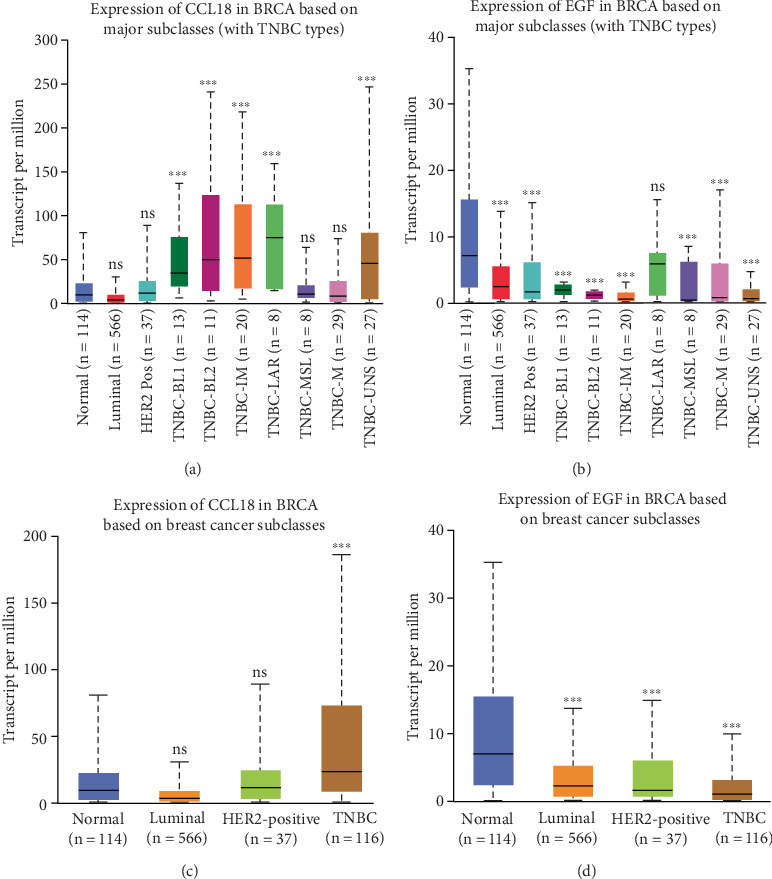
Differential expression of CCL18 and EGF across major breast cancer subtypes including triple-negative breast cancer (TNBC). This figure illustrates the expression profiles of CCL18 and EGF in breast invasive carcinoma (BRCA), stratified by molecular subtypes. (a, c) Boxplots depicting CCL18 expression levels across various clinical stages and molecular subtypes of breast cancer, including TNBC classifications (TNBC-BL1 (basal-like 1), TNBC-BL2 (basal-like 2), TNBC-IM (immunomodulatory), TNBC-M (mesenchymal), TNBC-MSL (mesenchymal stem-like), TNBC-LAR (luminal androgen receptor), and TNBC-UNS (unspecified)). (b, d) Boxplots displaying EGF expression under similar stratification. Statistical significance between groups was assessed, with *p* values indicated as follows: ns (not significant), *p* < 0.05 (∗), *p* < 0.01 (∗∗), and *p* < 0.001 (∗∗∗).

To further explore subtype-specific expression patterns, we assessed the expression of CCL18 and EGF across defined molecular subtypes of BRCA, including luminal A, luminal B, HER2-enriched, and triple-negative breast cancer (TNBC) (Figures 2a, 2b, 2c, and 2d). Notably, CCL18 expression was significantly elevated in TNBC cases compared to other subtypes and normal controls (Figure 2a,c), indicating a potential role for CCL18 in the aggressiveness and progression of this clinically challenging subtype. In contrast, EGF expression remained relatively unchanged across all subtypes, including TNBC, and did not demonstrate a significant association with tumor subtype or progression (Figure 2b,d).

These findings underscore a subtype-specific role for CCL18 in BRCA, particularly in TNBC, where its elevated expression may serve as a marker of tumor aggressiveness. Meanwhile, the minimal variation in EGF expression across stages and subtypes suggests its role in BRCA may be limited or context-dependent. Collectively, these data reinforce the potential utility of CCL18 as a biomarker for BRCA progression, while also delineating the differential relevance of CCL18 and EGF in the BRCA landscape.

### 3.3. Association of CCL18 and EGF Expression With Immune Cell Infiltration in BRCA

To explore potential functional implications of CCL18 and EGF expression in the tumor microenvironment, we next investigated their potential roles in modulating the tumor immune microenvironment. To this end, Spearman's correlation analyses were performed to assess the relationship between the expression levels of these genes and the infiltration of various immune cell populations in BRCA tissues from the TCGA dataset.

**Figure 3:**
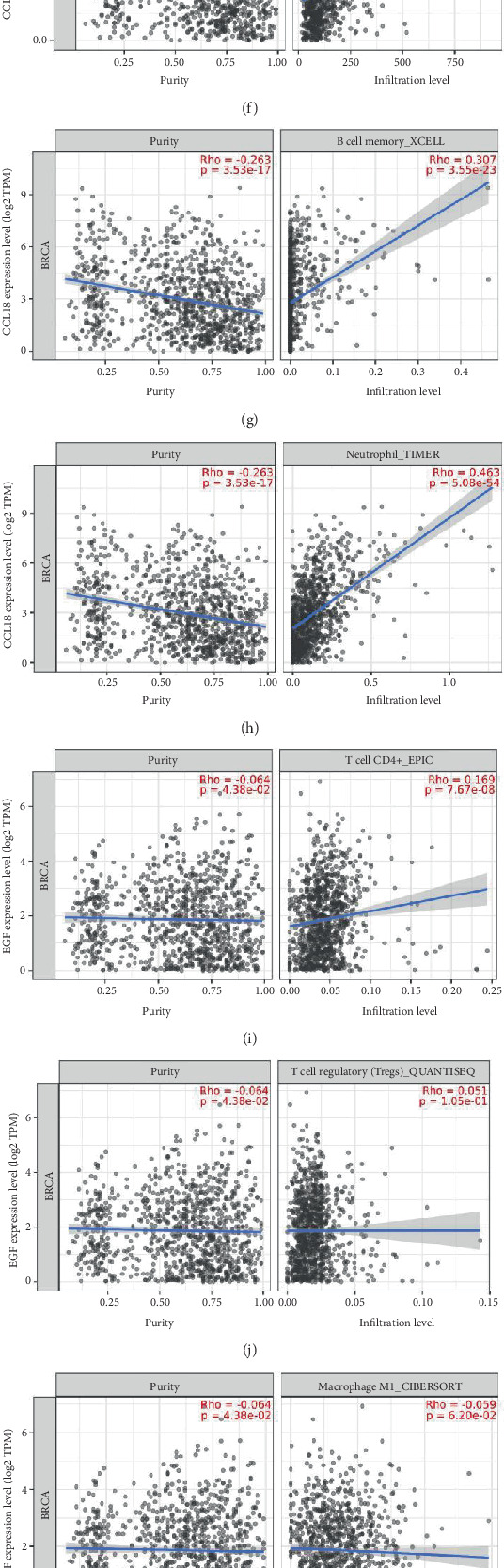
Correlation between CCL18 and EGF expression and immune cell infiltration in breast cancer. Scatter plot in the figure presents Spearman correlation analyses between gene expression and immune cell infiltration levels in breast cancer using the TIMER2.0 platform. (a–h) The correlation of CCL18 expression with various immune cell types: (a) CD4^+^ T cells, (b) regulatory T cells, (c) M1 macrophages, (d) M2 macrophages, (e) myeloid dendritic cells, (f) monocytes, (g) memory B cells, and (h) neutrophils. Similarly, (i–p) the relationship between EGF expression and immune infiltration, including (i) CD4^+^ T cells, (j) regulatory T cells, (k) M1 macrophages, (l) M2 macrophages, (m) myeloid dendritic cells, (n) monocytes, (o) memory B cells, and (p) activated neutrophils. All correlations were computed using transcriptomic data from TCGA breast cancer samples. Statistically significant associations were determined based on *p* values < 0.05.

CCL18 expression demonstrated significant positive correlations with a diverse array of immune cells, including CD8^+^ T cells (Figure 3a), regulatory T cells (Tregs, Figure 3b), both M1 (proinflammatory) and M2 (immunosuppressive) macrophages (Figure 3c,d), myeloid dendritic cells (Figure 3e), monocytes (Figure 3f), memory B cells (Figure 3g), and neutrophils (Figure 3h). These results suggest that CCL18 may influence the recruitment or activation of multiple immune cell subsets, potentially contributing to both immune surveillance and immune evasion mechanisms within the BRCA microenvironment.

Similarly, EGF expression was positively associated with several immune cell types, including CD8^+^ T cells (Figure 3i), Tregs (Figure 3j), M2 macrophages (Figure 3l), myeloid dendritic cells (Figure 3m), monocytes (Figure 3n), memory B cells (Figure 3o), and neutrophils (Figure 3p). In contrast, a negative correlation was observed between EGF expression and M1 macrophage infiltration (Figure 3k), indicating a potential skewing of the immune landscape toward a more immunosuppressive state in the presence of higher EGF levels.

These immune cell association patterns suggest that both CCL18 and EGF are engaged in complex immune regulatory processes within breast tumors. The strong positive correlations of CCL18 with both effector and suppressive immune cells reinforce its potential dual role in immune modulation. Meanwhile, the contrasting correlations of EGF—particularly its inverse relationship with M1 macrophages—highlight its likely contribution to an immunosuppressive tumor microenvironment.

Taken together with previous findings on their differential expression in BRCA and other malignancies, these data support the notion that CCL18 and EGF not only serve as markers of tumor biology but may also actively influence immune cell dynamics and tumor–immune interactions. Additional correlations with other immune regulatory factors are provided in Figure S2 (A–J), offering broader insight into the immunological impact of these genes in BRCA.

### 3.4. Prognostic Significance of CCL18 and EGF in BRCA and Other Cancers

Given the involvement of CCL18 and EGF in BRCA progression and their association with immune cell infiltration, we next evaluated their prognostic relevance by analyzing patient survival outcomes. Using the Kaplan–Meier plotter, we assessed the impact of gene expression levels on RFS and OS in BRCA patients.

**Figure 4:**
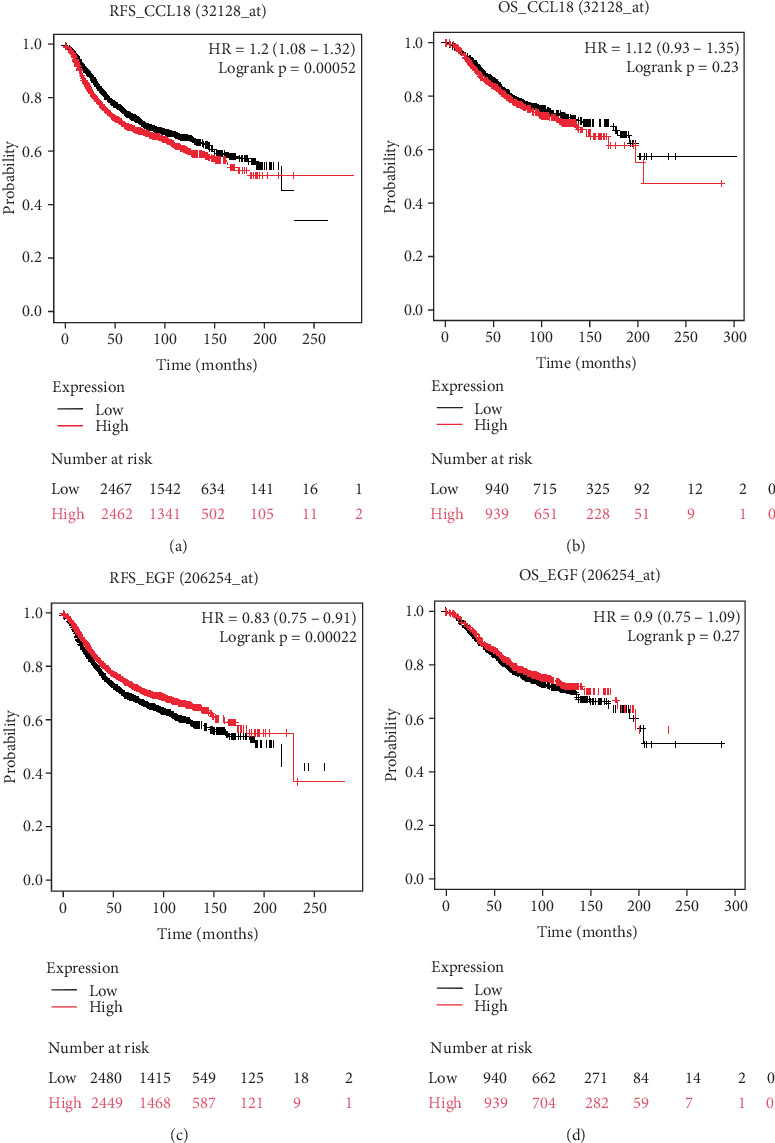
Prognostic impact of CCL18 and EGF expression on survival outcomes in BRCA patients. Kaplan–Meier survival analyses were conducted to assess the prognostic significance of CCL18 and EGF gene expression in breast invasive carcinoma. Survival curves compare high versus low expression levels of each gene for both relapse-free survival (RFS) and overall survival (OS). (a) The effect of CCL18 expression on RFS (*n* = 4929), while (b) its association with OS (*n* = 1879). (c, d) The correlation of EGF expression with RFS (*n* = 4929) and OS (*n* = 1879), respectively. Gene expression data were retrieved using valid Affymetrix probe IDs: 32128_at for CCL18 and 206254_at for EGF. Patients were stratified into high and low expression groups, with high-expression cohorts represented by red lines and low-expression cohorts by black lines. All analyses were performed using the Kaplan–Meier plotter tool (http://www.kmplot.com/), and statistical significance was evaluated to determine the impact of gene expression on patient survival outcomes.

For CCL18, survival analysis was conducted using the Affymetrix probe ID: 32128_at. Patients with low CCL18 expression exhibited significantly reduced RFS (*n* = 4929; HR = 1.20 [1.08–1.32]; *p* = 0.0005) (Figure 4a). However, no significant association was observed between CCL18 expression and OS (*n* = 1879; HR = 1.12 [0.93–1.35]; *p* = 0.23) (Figure 4b), suggesting that CCL18 may primarily influence early recurrence rather than long-term survival outcomes.

Conversely, EGF survival analysis was conducted using the Affymetrix probe ID: 206254_at and expression demonstrated an inverse trend. High EGF expression was significantly correlated with shorter RFS (*n* = 4929; HR = 0.83 [0.75–0.91]; *p* = 0.0002) (Figure 4c), while no significant effect was found on OS (*n* = 1879; HR = 0.90 [0.75–1.09]; *p* = 0.27) (Figure 4d). These findings indicate that both CCL18 and EGF are significantly associated with disease recurrence, though their impact on overall mortality may be influenced by additional clinical variables.

To determine whether these prognostic associations extend beyond BRCA, we conducted a similar analysis in colon cancer. Elevated CCL18 expression in colon cancer patients was significantly associated with both poorer RFS (*n* = 1336; HR = 1.33 [1.07–1.65]; *p* = 0.0099) and reduced OS (*n* = 1061; HR = 1.34 [1.09–1.65]; *p* = 0.0048) (Figures S3A and S3B). For EGF, lower expression levels were linked to poorer RFS (*n* = 1336; HR = 0.54 [0.54–0.86]; *p* = 0.0011) (Figure S3C), whereas high expression correlated with reduced OS (*n* = 1061; HR = 0.70 [0.56–0.88]; *p* = 0.0017) (Figure S3D). These observations suggest that CCL18 and EGF may serve as broader prognostic indicators across multiple cancer types.

**Figure 5:**
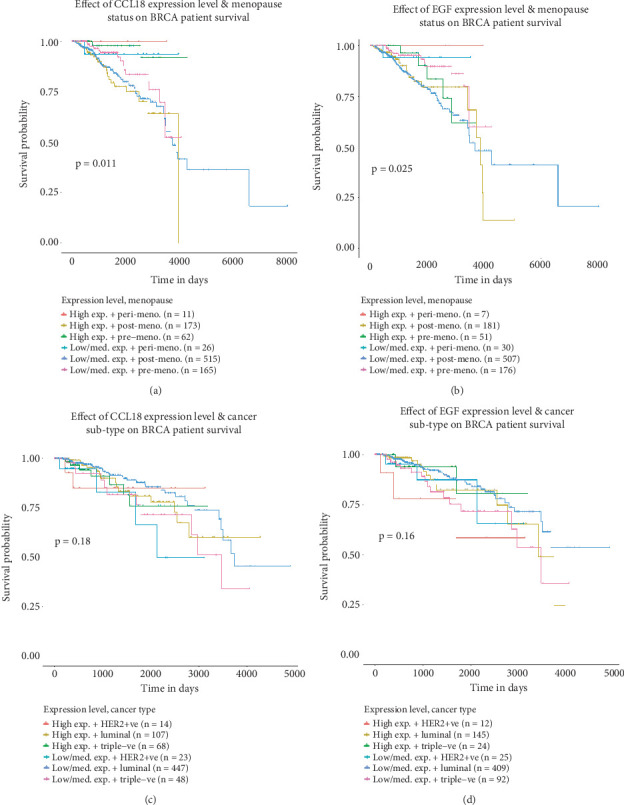
Survival analysis of CCL18 and EGF expression in relation to clinical features in breast cancer patients. Kaplan–Meier survival plots illustrate the association between CCL18 and EGF expression levels and clinical parameters in breast invasive carcinoma (BRCA) patients. (a, b) The impact of CCL18 and EGF expression, respectively, in relation to menopause status on patient survival. (c, d) Survival differences based on expression levels of CCL18 and EGF, respectively, across various breast cancer subtypes. The data were obtained from TCGA breast cancer cohorts and analyzed using the UALCAN web portal (http://ualcan.path.uab.edu/). Values with *p* < 0.05 were considered as significant.

The TCGA datasets were also analyzed to assess the survival probability of BRCA patients. Further stratification of BRCA patients by menopausal status using TCGA datasets revealed an additional layer of complexity. Patients in the postmenopausal group with high expression of both CCL18 and EGF exhibited significantly decreased survival probabilities compared to their low- and medium-expression counterparts (Figure 5a,b, *p* < 0.05). However, across the entire BRCA cohort, the survival differences between high and low/medium expression groups for both genes did not reach statistical significance (CCL18, *p* = 0.18; EGF, *p* = 0.16) (Figure 4c,d), highlighting the potential influence of menopausal status and hormonal milieu on gene-related prognosis.

In summary, these survival analyses underscore the prognostic potential of CCL18 and EGF in BRCA, particularly in relation to RFS. Their prognostic impact appears to be context-dependent, influenced by both tumor type and patient-specific variables such as menopausal status. Moreover, the extension of these associations to colon cancer supports their broader relevance in oncology and highlights their potential utility as biomarkers for cancer progression and patient risk stratification.

### 3.5. Promoter DNA Methylation Analysis of CCL18 and EGF in BRCA

To investigate potential epigenetic mechanisms underlying the aberrant expression of CCL18 and EGF in BRCA, we next investigated whether epigenetic alterations contribute to their aberrant expression. Specifically, we assessed the DNA methylation status of their promoter regions, as promoter hypermethylation often correlates with transcriptional repression, while hypomethylation may facilitate gene activation.

**Figure 6:**
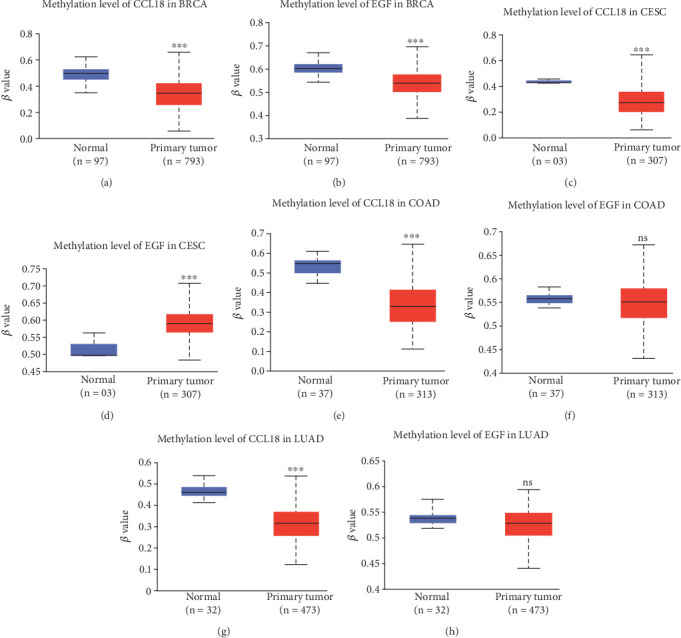
Comparative methylation and expression profiles of CCL18 and EGF in human cancers using TCGA data. Boxplots display the association between promoter DNA methylation and mRNA expression levels for the genes CCL18 and EGF across various cancer types, utilizing data from the TCGA database. (a) Methylation and expression trends for CCL18 in breast cancer, while (b) the same for EGF. (c) Methylation and expression data for CCL18 in cervical squamous cell carcinoma and (d) for EGF. In colon adenocarcinoma (COAD) samples, promoter methylation and expression levels for CCL18 are depicted in (e) and for EGF in (f). (g, h) Corresponding data for CCL18 and EGF, respectively, in lung adenocarcinoma samples. Statistical significance is indicated as follows: *p* < 0.05 (∗), *p* < 0.01 (∗∗), and *p* < 0.001 (∗∗∗), while “ns” signifies no statistically significant difference.

Using TCGA dataset, we analyzed promoter methylation levels of CCL18 and EGF in BRCA tissues compared to normal controls. DNA methylation was quantified using beta values, ranging from 0 (completely unmethylated) to 1 (fully methylated). The analysis revealed significantly reduced promoter methylation levels for both CCL18 and EGF in primary breast tumor samples relative to normal breast tissues (normal *n* = 97; tumor *n* = 793; *p* < 0.05) (Figure 6a,b). This widespread hypomethylation aligns with their elevated gene expression observed in previous sections, suggesting a potential epigenetic mechanism driving their transcriptional activation in BRCA.

To further elucidate the relationship between DNA methylation and clinical variables, we stratified methylation data based on nodal metastasis status, molecular subtypes, and menopausal status. Across all nodal stages (N0 to N3), promoter methylation levels of CCL18 and EGF remained significantly lower in tumor samples compared to normal tissue (normal: 97; N0 = 230; N1 = 242; N2 = 97; N3 = 20; *p* < 0.05) (Figures S4A and S4D). Similar patterns were observed across major BRCA subtypes, including luminal (*n* = 393), HER2-positive (*n* = 17), and TNBC (*n* = 84) (Figures S4B and S4E), with consistent hypomethylation compared to normal tissue (*p* < 0.05). Additionally, analysis stratified by menopausal status (premenopause *n* = 171, perimenopause *n* = 23, postmenopause *n* = 506) confirmed persistent promoter hypomethylation of both genes irrespective of hormonal status (Figures S4C and S4F).

To assess whether the identified epigenetic modifications are specific to BRCA or represent a broader oncogenic mechanism, we extended our analysis to include CESC, colon adenocarcinoma (COAD), and LUAD. Remarkably, CCL18 exhibited significant promoter hypomethylation not only in BRCA but also across cervical, colon, and lung cancer tissues (Figures 6, 6, and 6). This consistent pattern of hypomethylation suggests that aberrant epigenetic regulation of CCL18 may constitute a common feature across multiple malignancies.

In contrast, EGF displayed a tumor type–specific methylation profile. In cervical cancer, promoter methylation of EGF was markedly increased in tumor tissues compared to normal controls (normal *n* = 3; tumor *n* = 307), implying potential transcriptional silencing via epigenetic repression (Figure 6d). However, in both colon and lung cancers, EGF promoters were significantly hypomethylated relative to their respective normal tissues (Figure 6f,h), indicating a possible activation of EGF expression through loss of methylation. These findings underscore the context-dependent nature of epigenetic regulation in cancer and highlight the importance of tissue-specific mechanisms in gene expression control.

In summary, our DNA methylation analysis provides evidence of widespread hypomethylation of CCL18 and EGF promoters in BRCA compared to normal breast tissues, which may contribute to their upregulated expression. As the DNA hypomethylation is mostly linked to an increase in gene expression, the observed changes in DNA methylation, particularly in relation to disease stage, subtype, and menopausal status, further emphasize the role of epigenetic modifications in the regulation of these biomarkers in BRCA. Moreover, the comparison with other cancers like cervical squamous cell carcinoma, colon cancer, and lung cancer suggests that these methylation patterns may be specific to BRCA, pointing to a potential mechanism underlying the upregulation of CCL18 and EGF in breast tumorigenesis.

### 3.6. Construction and Analysis of Gene Interaction Networks for CCL18 and EGF

To better understand the functional networks surrounding CCL18 and EGF, we next sought to explore transcriptome-based gene interaction networks to identify coexpressed partners of CCL18 and EGF in BRCA. This approach provides systems-level insights into the functional connectivity and biological roles of these genes.

**Figure 7:**
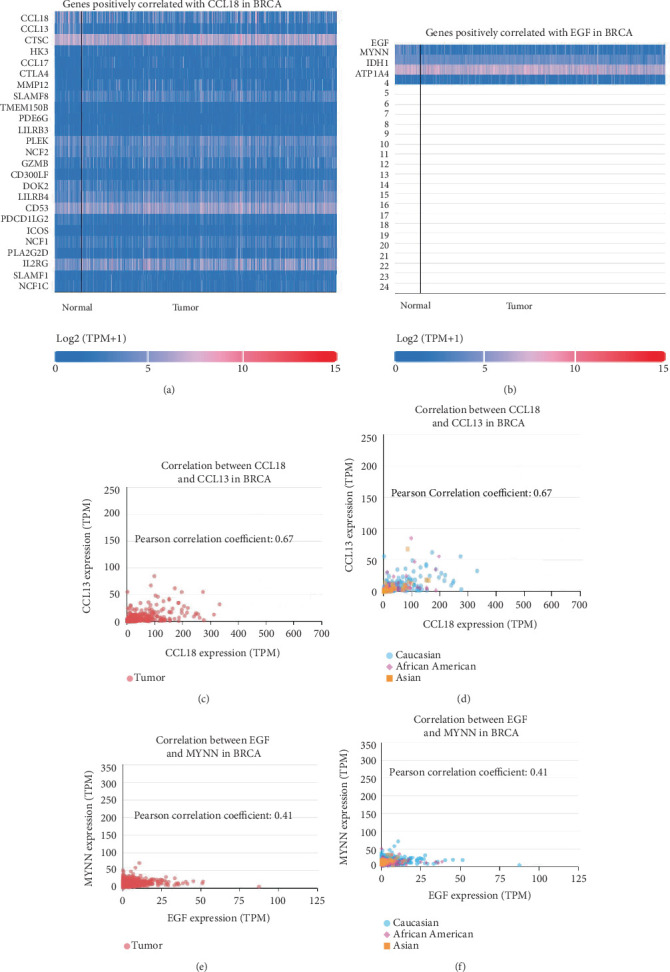
Correlation analysis of CCL18 and EGF gene expression in breast invasive carcinoma. This figure presents gene expression correlation analyses for CCL18 and EGF in breast invasive carcinoma using TCGA data. (a) Heat map displaying the top 25 genes with the strongest positive correlation to CCL18. (b) Heat map showing the top 3 genes with the highest positive correlation to EGF. (c) Representative image of all other genes. Scatter plot highlighting the gene with the highest Pearson correlation coefficient (*r* > 0.3) to CCL18 across all breast cancer samples. (d) Representative image of all other genes. Correlation plot for the same top CCL18-associated gene stratified by patient race. (e) Representative image of all other genes. Scatter plot illustrating the most positively correlated gene with EGF expression (Pearson correlation coefficient > 0.3) in breast cancer patients. (f) Representative image of all other genes. Corresponding correlation analysis for EGF in patients of different racial backgrounds. Gene correlation data were obtained from UALCAN (http://ualcan.path.uab.edu/), and the scatter plots visualize the strength and direction of expression relationships.

Using transcriptomic data from BRCA samples, we performed correlation-based network analysis to identify genes positively associated with CCL18 and EGF expression. For CCL18, the top 25 genes exhibiting strong positive correlations were identified (Figure 7a), while the top 3 genes most significantly correlated with EGF were also highlighted (Figure 7b). These gene sets likely reflect molecular partners or pathways modulated by CCL18 and EGF, encompassing processes such as immune regulation, extracellular matrix remodeling, and cell migration—hallmarks of cancer progression.

To further illustrate these relationships, we generated scatter plots showing the coexpression patterns between CCL18 or EGF and their top correlated genes (Figures 7c, 7d, 7e, and 7f). These visualizations confirm the strength and consistency of the observed associations and support the hypothesis that both CCL18 and EGF integrate into broader transcriptional programs relevant to tumor behavior.

Importantly, many of the genes coexpressed with CCL18 are implicated in immune-related functions, complementing our earlier findings that linked CCL18 expression with immune cell infiltration and TAM activity. In contrast, the limited but strong correlations observed for EGF suggest a more focused regulatory role, possibly related to growth factor–mediated signaling rather than widespread immune modulation.

Together, these interaction networks shed light on the system-level impact of CCL18 and EGF in BRCA biology. The coexpressed gene signatures not only reinforce the multifaceted functions of these genes but also present new candidates for functional validation and therapeutic exploration. By integrating gene expression, epigenetic, immune, and survival data, this analysis underscores the central roles of CCL18 and EGF within the tumor ecosystem and highlights the value of network-based approaches in uncovering novel regulatory axes in cancer progression.

### 3.7. PPI Networks of CCL18 and EGF

To complement transcriptomic coexpression findings, we next analyzed PPI networks to characterize the molecular interactions of CCL18 and EGF at the protein level. These networks offer mechanistic insights into how each protein engages in distinct signaling cascades relevant to BRCA progression.

For CCL18, the top 10 predicted interacting proteins, based on the STRING score, were PITPNM3 (score = 0.993), CCR8 (score = 0.988), CXCR5 (score = 0.871), CCR5 (score = 0.830), CCL19 (score = 0.830), CCR3 (score = 0.830), CCR1 (score = 0.825), CCR2 (score = 0.817), CCR7 (score = 0.789), and CD163 (score = 0.787) (Figure S5A). These interactions primarily suggest CCL18's involvement in immune response and chemotaxis, particularly in the context of macrophage and T cell recruitment to tumor sites, which correlates with its previously discussed role in BRCA progression.

In contrast, EGF demonstrated a broader and more robust network of functional protein associations. The top 10 predicted interacting proteins for EGF included EGFR (score = 0.999), ERBB2 (score = 0.993), ERBB3 (score = 0.992), PCSK9 (score = 0.989), P4HB (score = 0.989), ERBB4 (score = 0.987), GRB2 (score = 0.984), SHB (score = 0.969), F3 (score = 0.966), and FGF2 (score = 0.959) (Figure S5B). These interactions point to a more complex signaling network, predominantly involving the EGFR family and downstream signaling pathways critical for cell division, proliferation, and survival.

These interaction network analyses reinforce the distinct and complex roles of CCL18 and EGF in cancer biology, particularly in BRCA. While CCL18 predominantly contributes to immune cell signaling, EGF is deeply integrated into cellular signaling and proliferation pathways, making it a central player in tumorigenesis. These networks offer valuable insights into how these proteins interact with other cellular components to influence cancer progression.

## 4. Discussions

We investigated the relationship between the expression of the CCL18 and EGF genes using information from TCGA. The approach taken here is intended to examine substantial malignancies all across the complete genome to find genetic interconnections and linkages between solid carcinomas at different levels in contrast to normal surrounding tissues.

Previously, many scientists did not use data from computational biology or “in silico” techniques. The acceptance of these techniques has substantially expanded due to the quick development of machine learning strategies and bioinformatics tools. For logical drug design, 3D (three-dimensional) structures are also exceptionally valuable. Moreover, several sequence-based databases have been created as a result of the exponential increase in biological sequences found in the postgenomic age and their successful application in drug discovery [27]. Pharmaceutical science has undergone a spectacular transformation in a consequence of swift discoveries in RNA sequencing and molecular bioinformatics, in which computational biology is increasingly being used to promote the development of novel drugs [28]. Because of this, we analyzed the genetic correlation between the onset and progression of BRCA in this study using computational techniques and bioinformatics tools.

Our results suggests that CCL18 and EGF gene expression is significantly associated with the genesis, progress, and prevalence of BRCA risks. Additionally, our analysis revealed that patients with postmenopausal status and high levels of both CCL18 and EGF expression showed lower survival probabilities than those in other groups, as evaluated by analyzing the survival of TCGA datasets. In contrast, the survival ability of prostate cancer patients indicated that CCL18 is more potential than the EGF. Therefore, EGF has overall less survival ability in prostate cancer but is high in BRCA compared to CCL18. We also evaluated DNA methylation in several breast subtypes and found that all stages and subclasses of BRCA had significantly lower levels of DNA methylation in their CCL18 and EGF-expressing tissues compared to their corresponding normal breast tissues (*p* < 0.05). For the nodal metastatic stage, major subtypes, and menopause status, the supplemental data also revealed similar outcomes. This finding implies that DNA methylation may be one potential mechanism underlying the increased production of CCL18 and EGF in BRCA.

Additionally, except for macrophage M1 of EGF, the immune infiltration profile in BRCA disclosed a positive connection in all T cell CD4+, Treg, monocyte, memory B cell, and neutrophil cells that expressed CCL18 and EGF. Furthermore, CCL18 demonstrated a positive association with activated NK cells and the others in contrast to EGF. This correlation undoubtedly indicates that CCL18 is more susceptible to BRCA than EGF. The STRING database shows that the interaction network is most enriched in chromosome segregation, small GTPase-mediated signal transduction, and cell division in the cases of CCL18 and EGF, respectively. In contrast to CCL18, EGF was shown to have a considerable functional associate interaction. The majority of genes are part of networks that help them function rather than acting independently. To build and comprehend gene interaction/regulatory networks, expression-based gene correlations are particularly helpful.

After thorough analysis, we discovered the top 25 genes that are positively correlated with CCL18 (Figure 7a), but only three genes are positively correlated with EGF (Figure 7b). Most importantly, the Pearson correlation coefficient value between CCL18 and CCL13 gene expression is 0.67 in Figures 7c, 7d, 7e, and 6f, whereas it is only 0.41 for EGF and MYNN gene expression. CCL18, therefore, is more favorably connected to BRCA than EGF. CCL13 is a member of the CC chemokine family that regulates the chemotaxis of several immune cells. Despite substantial research into its role in several illnesses, a comprehensive analysis of CCL13 is not yet available. In particular, CCL13's role in cancer is comparatively well known. Several studies suggest that CCL13 is associated with 2-microglobulin (2-MG) levels in multiple myeloma and cell proliferation in BRCA [29, 30]. If it is possible to associate CCL13 and CCL18 genes with BRCA in future research, scientists may be able to establish a strong linkage between these two genes in BRCA.

## 5. Limitations

While our study provides comprehensive bioinformatics insights into the expression, prognostic significance, immune association, and epigenetic regulation of CCL18 and EGF in BRCA, several limitations should be acknowledged. Primarily, the findings are based entirely on in silico analyses using publicly available datasets and computational tools, without experimental validation. Although these methods offer robust statistical and predictive power, they cannot fully capture the biological complexity underlying gene regulation and cancer progression. A key limitation is the absence of functional assays to directly demonstrate the roles of CCL18 and EGF in tumor development and progression. Specifically, we did not perform siRNA-mediated knockdown experiments to assess the phenotypic consequences of suppressing CCL18 or EGF expression in BRCA cell lines. Such functional validation would provide stronger causal evidence supporting our hypothesis that these genes contribute to BRCA aggressiveness and could serve as therapeutic targets. Additionally, while we analyzed associations across various cancer types and BRCA subtypes, our findings may be influenced by cohort heterogeneity and sample size disparities within the TCGA dataset. Future studies incorporating wet-lab experiments and clinical samples will be essential to validate and extend our findings, enhancing their translational potential in BRCA diagnostics and therapeutics.

## 6. Conclusion

Our comprehensive analyses highlight CCL18 and EGF as important molecular players in BRCA, though with distinct patterns of involvement. CCL18 is significantly overexpressed in BRCA tissues, correlates strongly with various immune cell infiltrates, and shows clear associations with RFS, likely influenced by promoter hypomethylation. In contrast, EGF expression was not significantly elevated in BRCA, yet its expression still correlated with immune infiltration and RFS, suggesting a nuanced regulatory role. DNA methylation analysis revealed reduced promoter methylation for both genes, indicating epigenetic influence. Coexpression and PPI networks revealed CCL18's primary involvement in immune modulation, while EGF was embedded in proliferative signaling cascades. Collectively, these findings position CCL18 as a potential prognostic marker and therapeutic target, while highlighting the complex, context-dependent role of EGF in BRCA biology and patient outcome.

## Data Availability

The data of this study are available on request from the corresponding author.

## Nomenclature

TCGA: The Cancer Genome Atlas
GSCA: Gene Set Cancer Analysis
STRING: Search Tool for the Retrieval of Interacting Genes
CCL18: C-C motif chemokine ligand 18
EGF: epidermal growth factor
ACC: adrenocortical carcinoma
BLCA: bladder urothelial carcinoma
BRCA: breast invasive carcinoma
CESC: cervical squamous cell carcinoma and endocervical adenocarcinoma
CHOL: cholangiocarcinoma
COAD: colon adenocarcinoma
DLBC: lymphoid neoplasm diffuse large B cell lymphoma
ESCA: esophageal carcinoma
GBM: glioblastoma multiforme
HNSC: head and neck squamous cell carcinoma
KICH: kidney chromophobe
KIRC: kidney renal clear cell carcinoma
KIRP: kidney renal papillary cell carcinoma
LAML: acute myeloid leukemia
LGG: brain lower grade glioma
LIHC: liver hepatocellular carcinoma
LUAD: lung adenocarcinoma
LUSC: lung squamous cell carcinoma
MESO: mesothelioma
OV: ovarian serous cystadenocarcinoma
PAAD: pancreatic adenocarcinoma
PCPG: pheochromocytoma and paraganglioma
PRAD: prostate adenocarcinoma
READ: rectum adenocarcinoma
SARC: sarcoma
SKCM: skin cutaneous melanoma
STAD: stomach adenocarcinoma
TGCT: testicular germ cell tumors
THCA: thyroid carcinoma
THYM: thymoma
UCEC: uterine corpus endometrial carcinoma
UCS: uterine carcinosarcoma
UVM: uveal melanoma

## Supporting Information

Supporting Information Additional supporting information can be found online in the Supporting Information section. Figure S1. Expression of CCL18 and EGF based on different stages of breast cancer. Figure S2. Correlation of CCL18 and EGF expression with immune cell infiltration in breast cancer. Figure S3. Kaplan–Meier survival curves showing the overall survival and relapse-free survival in colon cancer. Figure S4. Promoter methylation status of CCL18 and EGF in breast cancer subtypes and stages. Figure S5. Protein–protein interaction network of CCL18 and EGF.
