# Supplementary material for: CCL18 and EGF May Serve as Potential Prognostic Biomarkers and Therapeutic Targets for Human Breast Cancer
Source: Int J Breast Cancer. 2025 Jul 14;2025:8856457. doi: 10.1155/ijbc/8856457 (PMC12279427; doi:10.1155/ijbc/8856457)
Supplement: Supporting Information — Additional supporting information can be found online in the Supporting Information section. Figure S1. Expression of CCL18 and EGF based on different stages of breast cancer. Figure S2. Correlation of CCL18 and EGF expression with immune cell infiltration in breast cancer. Figure S3. Kaplan–Meier survival curves showing the overall survival and relapse-free survival in colon cancer. Figure S4. Promoter methylation status of CCL18 and EGF in breast cancer subtypes and stages. Figure S5. Protein–protein interaction network of CCL18 and EGF. [file 8856457.f1.docx]

**Title: CCL18 and EGF may serve as a potential prognostic biomarker and therapeutic target for human breast cancer**

Sm Faysal Bellah^1*^, Fatema Akter Sonia^2^ and Md. Razowanul Ferdous^1,3^, Olanrewaju Ayodeji Durojaye,^4, 5^ and Md. Robiul Islam^1^

^1^Department of Pharmacy, Manarat International University, Dhaka-1341, Bangladesh

^2^Department of Pharmacy, Bangabandhu Sheikh Mujibur Rahman Science & Technology University, Gopalganj, 8100, Bangladesh

^3^Research and development division, The ACME Laboratories Ltd., Dhaka-1350, Bangladesh

^4^Drug Discovery and Biotechnology Unit, Lion Science Park, University of Nigeria, Nsukka, 410001, Nigeria.

^5^Department of Chemical Sciences, Coal City University, Emene, Enugu State, Nigeria.

**Short title: CCL18 and EGF in breast cancer**

**E-mail address:**

SFB- faysal_phku@yahoo.com

FAS- bsmrstufatema@gmail.com

MRF- razowanul@gmail.com

OAD-lanredurojaye@mail.ustc.edu.cn

MRI- ribadhonkhan@gmail.com

^*^Corresponding author:

**Dr. Sm Faysal Bellah**

Department of Pharmacy,

Manarat International University

Dhaka-1341, Bangladesh

Tel: +8801844775670, +8801913261838

E-mail: faysal_phku@yahoo.com

Orchid ID: https://orcid.org/0000-0002-8626-8547


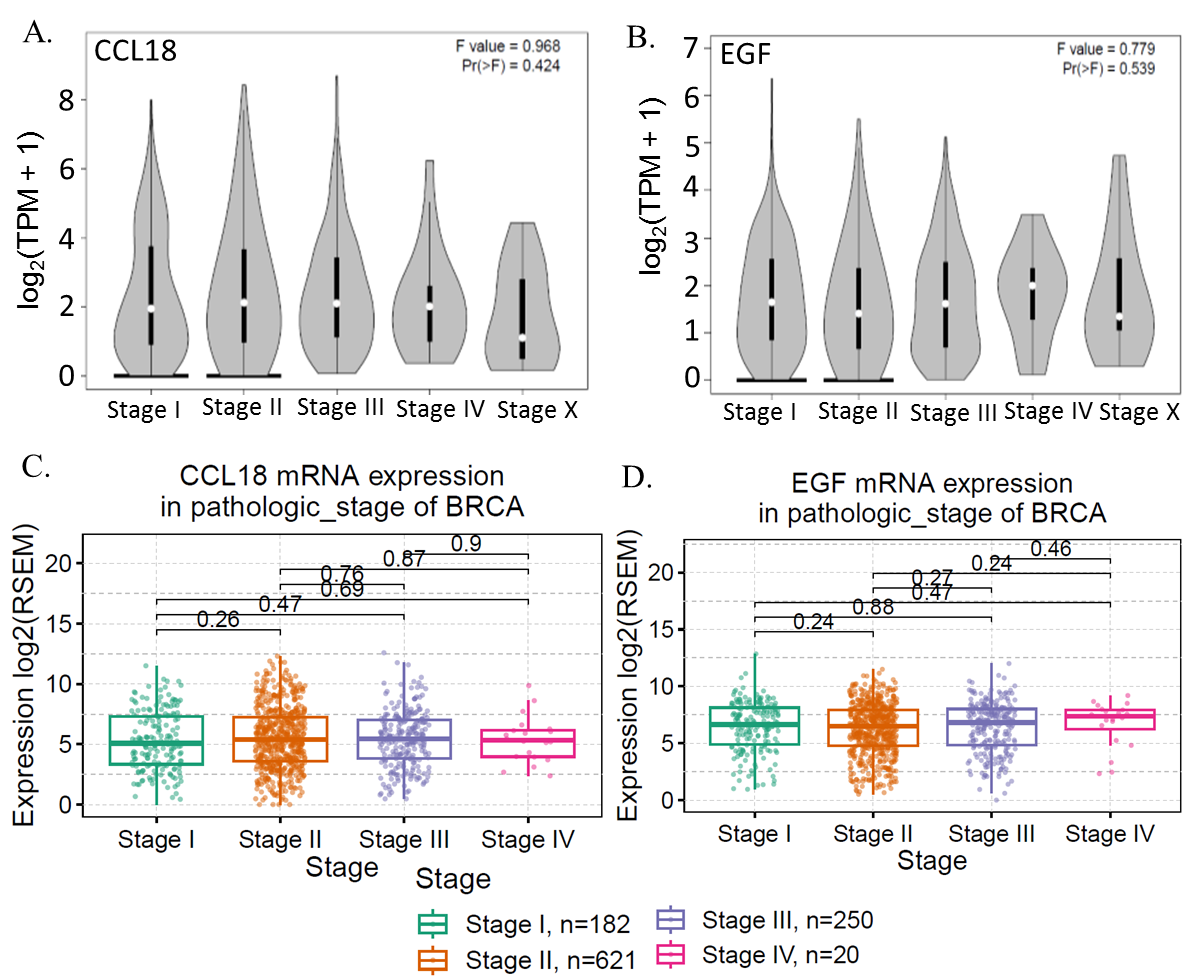


**Figure S1. Expression of CCL18 and EGF based on different stages of breast cancer.** Boxplots depict the expression levels of CCL18 (A) and EGF (B) across different pathological stages of breast cancer, as analyzed through the GEPIA2 platform using TCGA transcriptomic data. (C-D) Boxplots depict the expression levels of CCL18 (C) and EGF (D) across different pathological stages of breast cancer, as analyzed through the Gene Set Cancer Analysis (GSCA) platform using TCGA transcriptomic data. The *p* values < 0.05 were considered statistically significant.


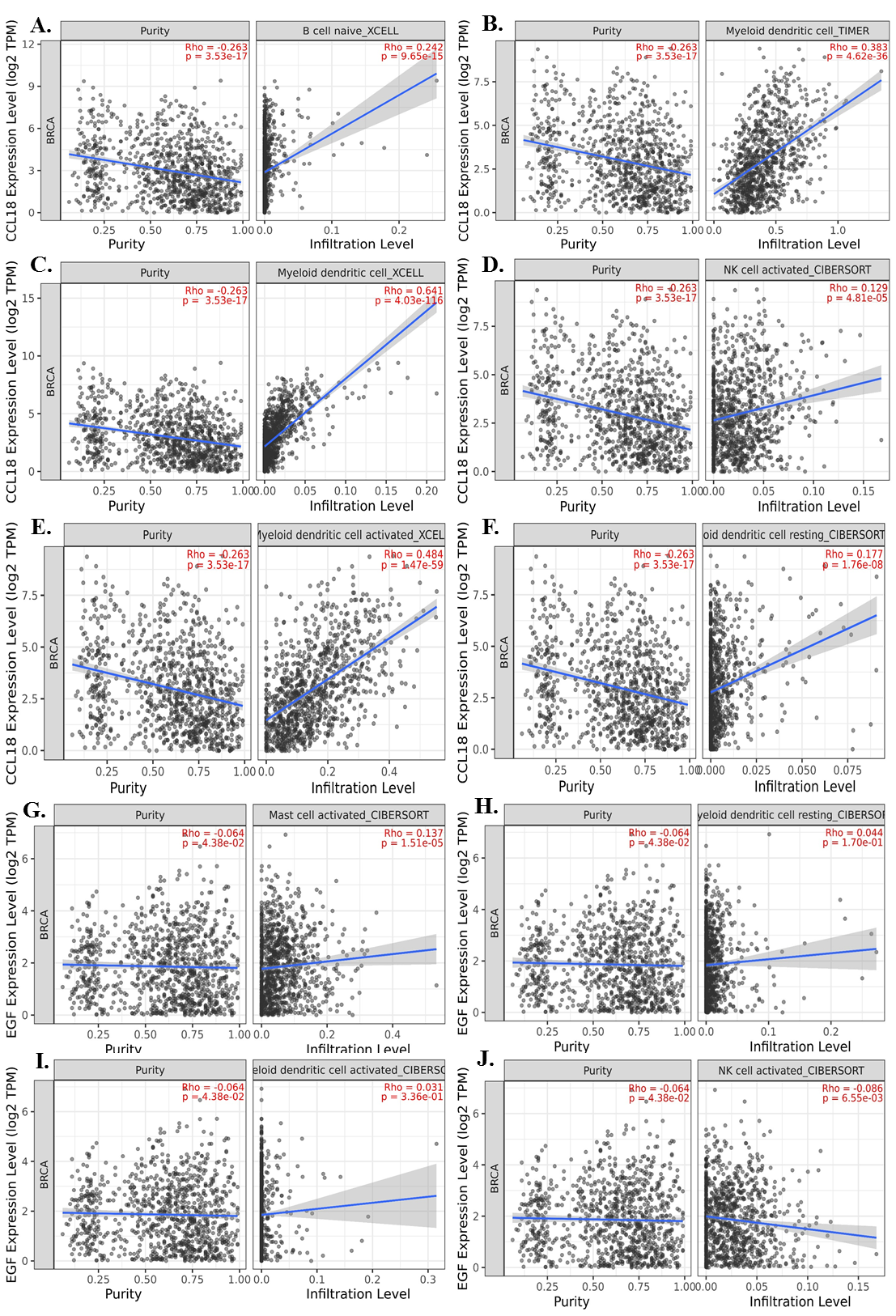


**Figure S2: Correlation of CCL18 and EGF expression with immune cell infiltration in breast cancer.** Scatter plots illustrating the correlation between gene expression and immune cell infiltration in breast cancer samples from the TCGA cohort, analyzed using TIMER2.0. (A–F) CCL18 expression showed significant positive correlations with multiple immune cell populations, including (A) naïve B cells, (B) myeloid dendritic cells, (C) myeloid dendritic cells (xCell), (D) activated NK cells, (E) activated myeloid dendritic cells (xCell), and (F) resting myeloid dendritic cells. (G–J) EGF expression was positively associated with (G) activated mast cells, (H) resting myeloid dendritic cells, and (I) activated myeloid dendritic cells, while a negative correlation was observed with (J) activated NK cells. All correlations were determined using spearman’s rank correlation, and p-values < 0.05 were considered statistically significant.


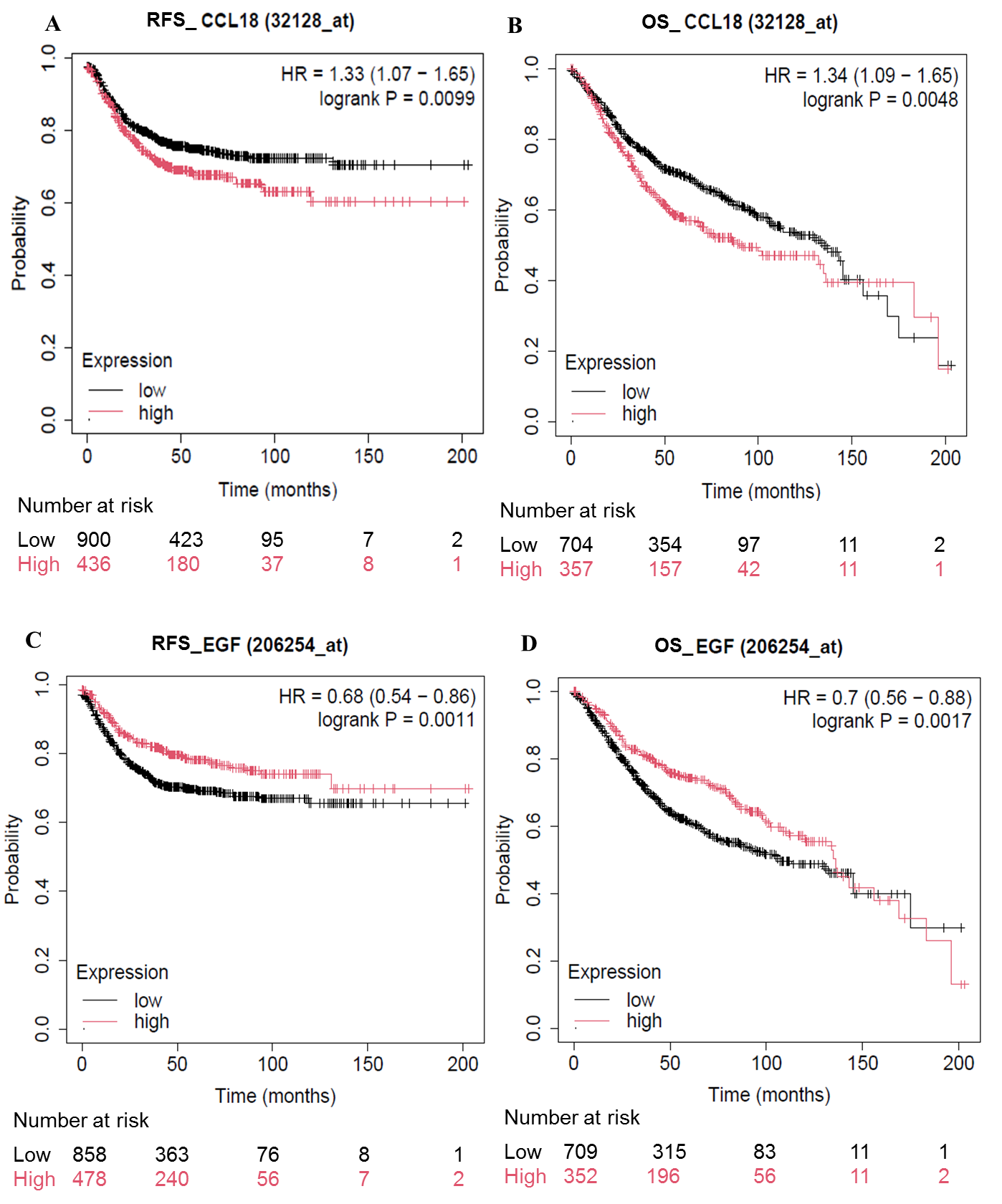


**Figure S3: Kaplan–Meier survival curves showing the overall survival and relapse free survival in colon cancer.** Survival curves were plotted with all breast cancer patients. (A) Effects of CCL18 with Relapse Free Survival (RFS, n= 1336) (B) Effects of CCL18 with Overall Survival (RFS, n= 1336) (C) Effects of EGF with Relapse Free Survival (OS, n=1061) and (D) Effects of EGF with Overall Survival (OS, n=1061). The desired Affymetrix ID was valid: 32128_at (CCL18) and 206254_at (EGF). OS and RFS were compared between CCL18 and EGF high-expression levels and low-expression level patients respectively. Red line represents tumours expressing high levels of CCL18 and EGF transcripts while the black lines represents tumours with low level CCL18 and EGF transcript expression. Data were analyzed using Kaplan–Meier plotter (www.kmplot.com).

**
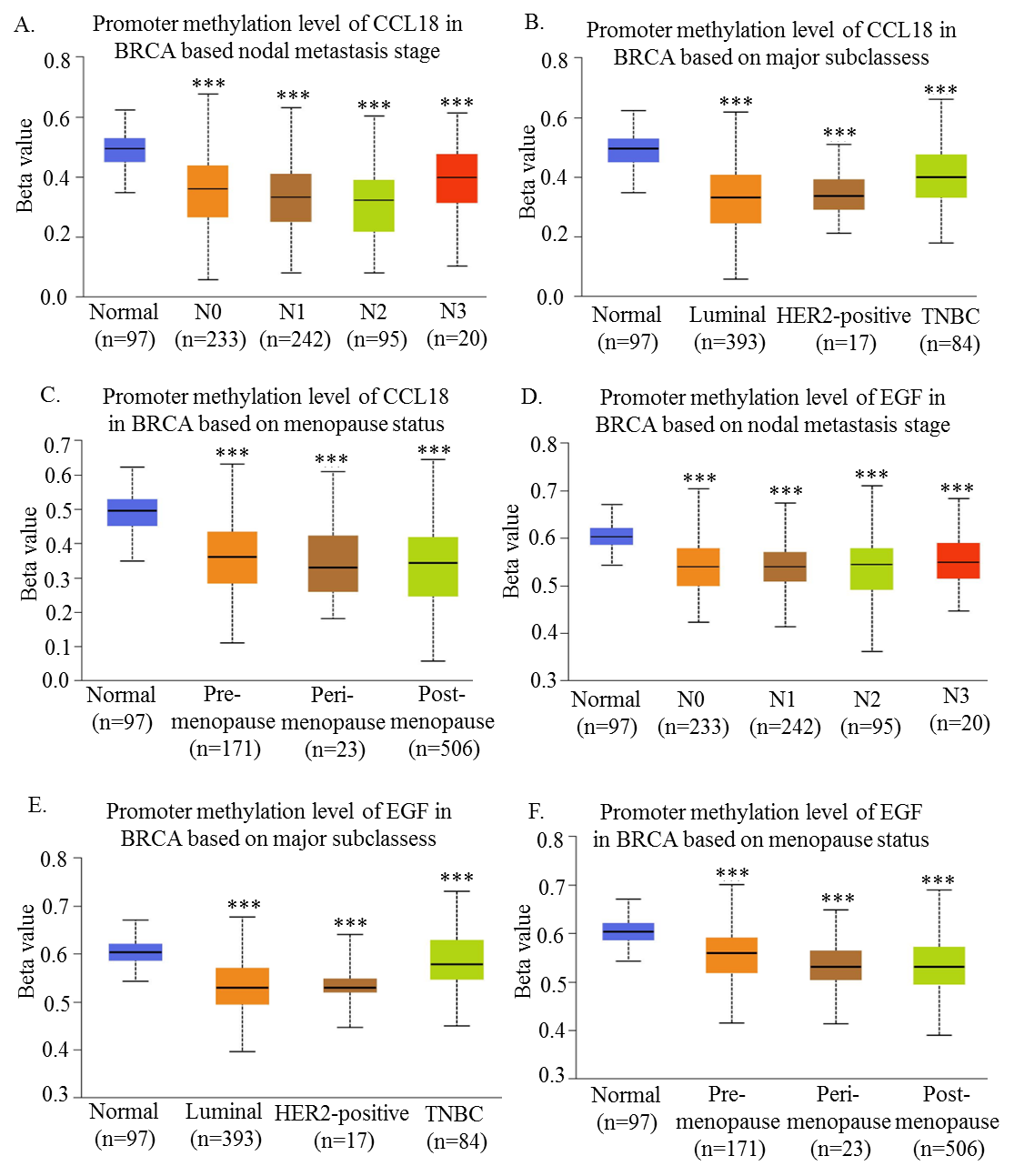
**

**Figure S4. Promoter methylation status of CCL18 and EGF in breast cancer subtypes and stages.** Boxplots illustrate the promoter DNA methylation patterns of CCL18 (A–C) and EGF (D–F) across various clinical stages and molecular subtypes of breast cancer. The analysis was performed using TCGA datasets via the UALCAN platform. Panels A–C display methylation levels of CCL18 in relation to breast cancer stage, molecular subtype, and clinical status, while panels D–F show corresponding methylation profiles for EGF. Statistical significance is denoted as follows: ns (not significant), p < 0.05 (*), p < 0.01 (**), and p < 0.001 (***).


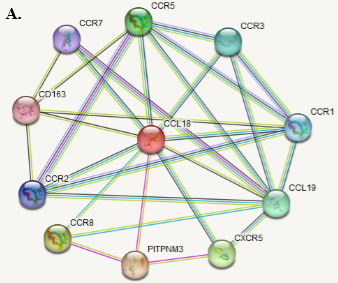

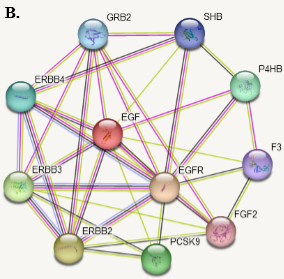


**Figure S5. Protein-Protein Interaction Network of CCL18 and EGF.** The protein-protein interaction networks for CCL18 (A) and EGF (B) were constructed using the STRING database. These networks illustrate the potential functional relationships and interaction partners of CCL18 and EGF in cellular processes. Abbreviations: CCL18, C-C Motif Chemokine Ligand 18; EGF, Epidermal Growth Factor; STRING, Search Tool for the Retrieval of Interacting Genes.
